# Probabilistic Human Health Risk Assessment of Heavy Metal Intake via Vegetable Consumption around Pb/Zn Smelters in Southwest China

**DOI:** 10.3390/ijerph16183267

**Published:** 2019-09-05

**Authors:** Guanghui Guo, Degang Zhang, Yuntao Wang

**Affiliations:** 1 Institute of Geographic Sciences and Natural Resources Research, Chinese Academy of Sciences, Beijing 100101, China (D.Z.) (Y.W.); 2 University of Chinese Academy of Sciences, Beijing 100049, China

**Keywords:** heavy metal, vegetable, dietary intake, probabilistic risk assessment

## Abstract

Vegetable contamination in mining and smelting areas has resulted in high dietary intakes of heavy metals, which pose potential health risks to local residents. In this study, paired soil-vegetable samples were collected around Pb/Zn smelters in Southwest China. Probabilistic risks to local residents via vegetable consumption were evaluated with a Monte Carlo simulation. The mean concentrations of As, Cd, Cu, Pb, and Zn in the soils were 116.76, 3.59, 158.56, 196.96, and 236.74 mg/kg, respectively. About 38.18%, 58.49%, and 52.83% of the vegetable samples exceeded the maximum allowable concentrations for As, Cd, and Pb, respectively. The daily dietary intake of As, Cd, and Pb exceeded the provisional tolerable daily intakes for local residents, with children showing the highest intake via vegetable consumption. The percentages of the target hazard quotients of As, Cd, and Pb for local residents exceeding the safe value of one were about 95%, 50%, and 25%, respectively. The 95th percentiles of the hazard index for children, adolescents, and adults were 15.71, 11.15, and 9.34, respectively, indicating significant risks to local residents, especially children. These results highlight a need to develop effective strategies to reduce heavy metal contamination and exposure to protect human health.

## 1. Introduction

Concern about soil contamination with heavy metals around the world has increased in recent decades because of dramatic social and economic development [1,2]. Various anthropogenic activities, such as mining and smelting [3,4], electrical waste dismantling [5], and agricultural practices [6], discharge heavy metals into the environment, resulting in elevated heavy metal concentrations in soils. According to the National Soil Pollution Survey Bulletin, 19.4% of farmland sampling sites in China are contaminated with heavy metals [7]. Zeng et al. [8] reported that 24.1% and 9.2% of vegetable field samples exceeded their corresponding thresholds for Cd and As, respectively. Vegetables grown in contaminated areas can readily accumulate heavy metals through root and foliar absorption [9]. Vegetable contamination with heavy metals around industrial areas has been found in China [10,11,12], Sweden [13], France [14], and Germany [15].

Vegetables are an important component of the human diet because they are rich in essential nutrients, and vegetable consumption is regarded as one of the main pathways of dietary exposure to heavy metals [16]. Epidemiological investigations have documented that chronic exposure to As, Cd, and Pb, even at trace levels, is associated with many diseases, such as dermal, cardiovascular, neurological, bone diseases, and cancers [17,18,19]. It is reasonable to infer that humans living near contaminated areas may have high health risks from heavy metal exposure. For example, the Itai-Itai disease in Japan was caused by consumption of rice cultivated in soils contaminated with Cd [20]. In addition, the presence of carcinogenic heavy metals such as Cd, Cr, and Pb in soils, fruits, and vegetables could be related to the high prevalence of cancer in Van Province, Turkey [21]. Dietary intakes of heavy metals are affected by dietary habits, heavy metal concentrations, and ingestion rates of foodstuffs for different age groups. Therefore, it is necessary to investigate the heavy metal concentrations in soils and vegetables, and then assess the dietary intakes and associated human health risks to different populations.

Previous studies have applied the conventional deterministic risk assessment method to evaluate human health risks via food consumption [22,23,24,25,26]. The mean or median value has frequently been used for exposure analysis in deterministic risk assessments, leading to over- or underestimation of the human health risk. In fact, uncertainty is unavoidable in these human health risk assessments because of the variabilities in the input variables. Fortunately, probabilistic risk assessment can effectively characterize uncertainties using probability distributions of the input variables in risk models [27]. A Monte Carlo simulation has been performed to describe the probability distributions of input variables and then estimate the probability of the risk that a hazard will occur [28,29]. In addition, sensitivity analysis in probabilistic risk assessment could be quantified via Monte Carlo simulation. Therefore, a comprehensive investigation involving a contamination assessment of heavy metals in soil and vegetable samples and an associated probabilistic health risk assessment should be conducted to provide more information on heavy metal control for environmental managers.

As the major industrial city in Yunnan Province, Gejiu is well known as the “tin capital of the world”. It has a long history of more than 200 years of non-ferrous metal mining and smelting. Wastewater, atmospheric dust, and tailings associated with mining and smelting activities are responsible for contamination of surrounding areas with heavy metals. Previous studies have reported As, Cd, Pb, and Zn concentrations in agricultural soils in Gejiu of 3.87, 5.84, 2.07, and 1.07 times their corresponding risk screening values, respectively [30]. However, few studies have systematically investigated paired soil–vegetable sample contamination and adverse effects on different populations via vegetable consumption [31,32].

The objectives of this study were to (1) qualify the concentrations of heavy metals in soils and vegetables surrounding Pb/Zn smelters, (2) compare the bioaccumulation ability of heavy metals in different vegetables, and (3) assess the dietary intakes and probabilistic health risks of heavy metals to different populations.

## 2. Materials and Methods

### 2.1. Study Area

**Figure 1:**
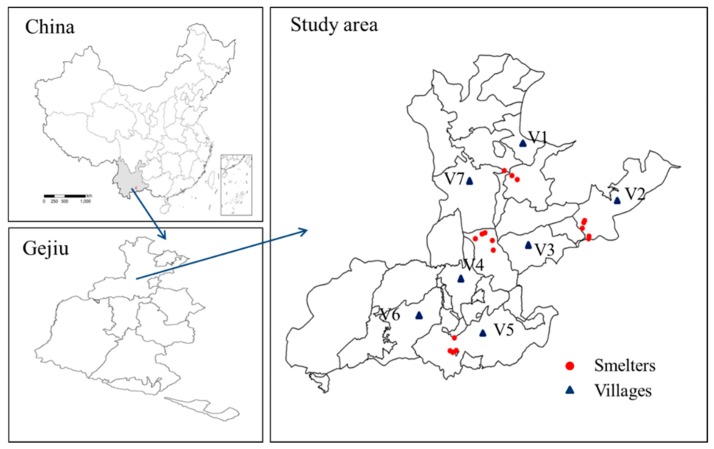
Location of sampling villages affected by smelting activities in suburban of Gejiu, China.

The study was carried out around Pb/Zn smelters situated in the suburban area in the northwest of Gejiu, China (Figure 1). Seven villages (V1, V2, V3, V4, V5, V6, and V7) were chosen as the study areas. These villages were far from the highways, and the traffic volumes in the road near the villages were relatively low. The straight-line distance between the village and Pb/Zn smelter were about 3–5 km. Many Pb/Zn smelters are gathered in the northwest suburban of Gejiu. Among the Pb/Zn smelters, Jijie smelter was constructed in 1958, which produces about 15,000 t Pb, 1000 t Zn, and 5000 t Sn every year [31]. A combination of cyclone and electrostatic precipitators is applied in the smelter in order to remove the particulates. In addition, wastewater disposal facilities have also been used for sewage purification. However, during the smelting process, a large amount of As, Pb, and Cd is still discharged into the surrounding environment via atmospheric deposition and wastewater discharge. Therefore, the agricultural soils around the smelters have been contaminated with heavy metals via atmospheric deposition and wastewater irrigation from the Pb/Zn smelters.

This area has a tropical monsoon climate, with an annual average temperature of 16.4 °C and annual precipitation of 1293 mm. The primary wind direction is southwesterly. V1, V2, V4, and V5, downwind of the prevailing wind, have mainly been contaminated by atmospheric deposition from the smelters. V3, V6, and V7 have mainly been contaminated by wastewater irrigation and atmospheric deposition. The soils in the study area are categorized as red clay soils according to the soil taxonomy system of China. The major agricultural products in the study area are vegetables and corn. In recent years, a large area of farmland around the Pb/Zn smelters has been used to cultivate vegetables for local residents.

### 2.2. Survey by Questionnaire

A total of 87 volunteers, including 25 children (4–11 years old), 26 adolescents (12–17 years old), and 36 adults (18–60 years old), completed a questionnaire in July 2017. All volunteers were native-born and were from seven villages around Pb/Zn smelters. Their ages, body weight, daily vegetable intakes, and residence periods were obtained in this survey. The informed consent from the participants has been obtained before the survey was conducted.

### 2.3. Soil and Vegetable Sampling

Seven villages were chosen as the study areas (Figure 1). From the seven villages, paired soil–vegetable samples (*n* = 97) were collected from the study area during July and August 2017. Five sub-samples were collected at each site with stainless shovels and mixed into one composite soil or vegetable sample. At each sampling site, approximate 500 g of the topsoil (0–20 cm depth) or 500 g of the relevant vegetable were collected. All the soil and vegetable samples were stored in self-sealing polyethylene bags and then transported to the laboratory as soon as possible. Vegetable samples were classified as leafy, rootstalk, legume, and fruit vegetables. The leafy vegetables included five pakchoi (*Brassica chinensis* L.), five spinach (*Spinacia oleracea* L.), six Chinses leek (*Allium tuberosum* R.), five celery (*Apium graveolens* L.), five garlic sprout (*Allium sativum* L.), five lettuce (*Lactuca sativa* L.), and five cabbage (*Brassica oleracea* L.) samples. The rootstalk vegetables included four ipomoea (*Ipomoea batatas* L.), five carrot (*Daucus carota* L.), five potato (*Solanum tuberosum* L.), four celery (*Apium graveolens* L.), six onion (*Allium cepa* L.), and four white radish (*Raphanus sativus* L.) samples. The legume vegetables included six soybean (*Glycine max* L.), five cowpea (*Vigna unguiculata* L.), five pea (*Pisum sativum* L.), and six kidney bean (*Phaseolus vulgaris* L.) samples. The fruit vegetables included six tomato (*Lycopersicon esculintum* M.), four eggplant (*Solanum melongena* L.), five cauliflower (*Brassica oleracea* L.), and six green pepper (*Capsicum annuum* L.) samples.

### 2.4. Soil and Vegetable Sample Preparation

Soil samples were air-dried at room temperature (25 °C), and then coarse materials and debris were removed. The soils were sieved through a 2.0-mm nylon sieve, and then further ground with a stainless steel grinder to pass through a 0.15-mm sieve. The ground soil samples were digested according to the U.S. Environmental Protection Agency (USEPA) method 3050B [33]. Briefly, 0.5 g of each soil sample was digested with 15 mL of ultrapure acid (HNO_3_/H_2_O_2_ = 2/1, *v*/*v*) in a digestion tube. Each digested sample was then heated on an electric hot plate at 120 °C to obtain 0.5 mL of a colorless solution. After cooling, the digested solutions were carefully filtered into separate volumetric flasks and diluted to 50 mL with ultrapure water.

The vegetable samples were washed with tap water three times and then rinsed with ultrapure water. The vegetable samples were oven-dried at 50 °C for 2–3 d to a constant mass and then ground using a stainless steel grinder. The fresh masses of the vegetables were recorded after absorbing the residual water. Each ground vegetable sample (1.0 g) was mixed with 10 mL of HNO_3_/HClO_4_ (9/1, *v*/*v*) in a digestion vessel overnight and then heated at 120 °C on an electric hot plate. The digested solutions were diluted with ultrapure water to 50 mL in volumetric flasks.

### 2.5. Chemical Analysis

The soil pH was measured in a soil/water solution with a ratio of 1:2.5 (*w*/*v*) using a pH meter (Mettler Toledo, 28-Standard, Five Easy Plus TM, Zurich, Switzerland) and soil organic matter was determined using the Walkley–Black method [34]. The concentrations of Cd, Cu, Pb, and Zn in soil and vegetable samples were determined by inductively coupled plasma mass spectrometry (ELAN DRC-e, Perkin Elmer SCIEX, Waltham, MA, USA), and the concentrations of As were analyzed by an atomic fluorescence spectrometer (KCHG AFS-9800 Beijing Haiguang Instrument, Beijing, China). The detection limits were 0.2, 0.03, 0.5, 0.4, 2.0, and 3.0 mg/kg for As, Cd, Cu, Ni, Pb, and Zn, respectively.

### 2.6. Quality Control Analysis

To ensure the accuracy and precision of the determination, duplicate samples, reagent blanks, and standard reference materials (GBW 08303 for soils and GBW 10015 for vegetables) were analyzed with each digestion batch. The recoveries of heavy metals in the samples ranged from 93% to 106%, and the relative standard deviations were less than 5%.

### 2.7. Data Analysis

#### 2.7.1. Concentrations Calculation

The concentrations of heavy metals in vegetables on the basis of fresh weight (*fw*) are calculated as follows:

(1) $$C_{veg\left(fw\right)}=C_{veg\left(dw\right)}\times \;\left(1-w\right)$$

where *C_veg_*_(*dw*)_ is the concentration of heavy metals in vegetables in dry weight, *C_veg_*_(*fw*)_ is the concentration of heavy metals in vegetables in fresh weight. *w* is the water content in vegetables. *w* of each vegetable sample was measured according to the Equation (2):

(2) $$w=(\frac{fresh\;mass-dry\;mass\;}{fresh\;mass})$$

where the fresh masses and dry masses of each vegetable sample were recorded during the cleaning and drying procedure.

#### 2.7.2. Bioaccumulation Factors

The bioaccumulation factors (BCFs) of the heavy metals were calculated using Equation (3), which can be used to evaluate the translocation abilities of the heavy metals from soil to the edible parts of the vegetables.

(3) $$\mathrm{BCF}=\frac{C_{veg}}{C_{soil}}$$

where *C_veg_* is the concentration of heavy metal in the vegetable (mg/kg, *fw*), and *C_soil_* is the concentration of heavy metal in the soil (mg/kg, *dw*).

#### 2.7.3. Exposure Assessment

Exposure to heavy metals via vegetable consumption is expressed as the estimated daily intake (EDI). The EDIs of the heavy metals were calculated using the following equation (4) proposed by the USEPA [35]:

(4) $$\mathrm{EDI}=\frac{EF\times ED\times C_{veg\;\left(fw\right)}\times IR\;}{BW\times AT}\;$$

where EDI is the estimated daily intake of the heavy metal (mg/(kg·day)), *EF* is the exposure frequency (day/year), *ED* is the exposure duration (year), *C_veg_*_(*fw*)_ is the concentration of the heavy metal in the vegetable (mg/kg, *fw*), *IR* is the daily ingestion rate of vegetables for the studied population (g/d), *BW* is the average body weight (kg), and *AT* is the average exposure time for non-carcinogenic effects (*ED* × 365 day/year). *EF*, *ED*, *IR*, and *BW* for different populations were obtained from the questionnaire in this study.

#### 2.7.4. Non-Carcinogenic Risk Characterization

Target hazard quotients (THQs) were calculated using Equation (5) to assess the non-carcinogenic risks of individual heavy metals to local residents [36]. Moreover, hazard indexes (HIs) were calculated as the sum of THQs of individual heavy metals using Equation (6) [36]. The HI represents the potential non-carcinogenic risk from multiple heavy metals.

(5) $$\mathrm{THQ}=\frac{ADI}{RfD}$$

(6) $$\mathrm{HI}=\overset{n}{\underset{i=1}{\sum }}THQ_{i}$$

where *RfD* is the chronic reference dose of heavy metals via ingestion exposure (μg/(kg *BW*·d)). The *RfD* values for As, Cd, Cu, Pb, and Zn were 0.3, 1, 40, 3.57, and 300 μg/(kg *BW*·d), respectively [37]. If the THQ or HI is ≤1, there is no obvious adverse effect on human health. Conversely, non-carcinogenic risks are likely to occur if the THQ or HI is >1.

#### 2.7.5. Monte Carlo Simulation

The probabilistic non-carcinogenic risk assessment of heavy metal exposure via vegetable consumption was performed using a Monte Carlo simulation. Weak correlation or independence between the input variables was assumed in this simulation. Input variables (*C_veg_*_(*fw*)_, *EF*, *ED*, *IR*, and *BW* in Equation 2) were modeled as specific probability distribution functions (Table S1), and the *RfD* was modeled as a point estimate because of a lack of sufficient toxicological data for each heavy metal. To ensure the reliability of the results, 30,000 random iterations of each input variable were carried out in each simulation. The input variables (i.e., *C_veg_*_(*fw*)_, *EF*, *ED*, *IR*, and *BW*) were randomly extracted from the defined probability distributions (Table S1). The probability distribution of the non-carcinogenic risk was generated when the probability distributions of the input variables were introduced to the risk assessment. In this study, the 5th, 25th, 50th, 75th, and 95th percentiles of the THQs for each heavy metal were extracted from the THQ probability distribution. The median, mean, and 95th percentiles of the HI were determined to estimate the non-carcinogenic risk of multiple heavy metals.

In addition, sensitivity analysis was performed to identify variables that affected the distribution of risk outcome and calculate the contribution of each input variable to the probabilistic health risk. The Spearman rank correlation was calculated for each input variable and the results are illustrated in tornado plots [38].

#### 2.7.6. Statistical Analysis

SPSS Statistics 22.0 (IBM Corp, Armonk, NY, USA) was used for statistical analysis, including analysis of variance, correlation analysis, and principle component analysis. Origin 2019 (Origin Lab Corp, Northampton, MA, USA) was used for distribution tests and charting. The Monto Carlo simulation was performed using Crystal Ball Software (11.1 Oracle Inc., Oracle, CA, USA).

## 3. Results and Discussion

### 3.1. Heavy Metal Concentrations in the Soils

Soil pH, soil organic matter, and heavy metal concentrations in the soil samples from the study areas are shown in Table 1. The soil pH range was 5.56–7.26 (mean 6.43), indicating that the soils in the study area were weakly acidic. The mean organic matter concentration in the soils was 45.47 g/kg and the range was 28.31–70.45 g/kg. As, Cd, Cu, Pb, and Zn concentrations [range (mean)] in the soil samples were 17.24–165.42 (116.76), 0.38–11.40 (3.59), 15.21–349.87 (158.56), 23.24–609.49 (196.96), and 16.21–939.83 (236.74) mg/kg, respectively. Compared with Chinese risk screening values (GB15618-2018), 51.67%, 100%, 53.87%, 62.5%, and 52.25% of the soil samples exceeded their corresponding risk screening values for As, Cd, Cu, Pb, and Zn, respectively.

The CV values ranged from 54.58% to 117.50% for As, Cd, Cu, Pb, and Zn, indicating strong variation of these heavy metals. Elevated concentrations coupled with high CV values indicated that serious contamination with these heavy metals in the study areas was caused by anthropogenic activities. The results of the Pearson correlation showed the significantly positive correlation between the As-Cd (0.981), As-Cu (0.577), Cu-Cd (0.525), and Pb-Zn (0.929) (*p* < 0.001) (Table S2), indicating the common sources for heavy metals. Principle components analysis further identify the possible sources for heavy metals in the soils (Table S3). Two components with eigenvalues > 1 were extracted and accounted for the 85.87% of the total variation. The first component (PC1) was dominated by Pb, Cd, Zn, and partially As, which explained 55.45% of the total variation. PC1 may be regarded as anthropogenic component associated with mining and smelting activities. As well known, there were many Pb/Zn and Sn smelters in the study area, and a large amount of heavy metals were discharged into the surrounding environment. The second component (PC2) included Cu and partially As, accounting for 30.42% of the total variation. PC2 may be considered as anthropogenic component related with agricultural activities. Pesticides containing As and Cu were usually applied during the vegetable growth, leading to the elevated concentrations of As and Cu in the soils. Previous studies have reported that pesticides consist of As and Cu [39,40]. In addition, livestock manure has high concentrations of As and Cu due to the feedstuff with As and Cu [41,42], which may be an important source of As and Cu in the agricultural soils.

### 3.2. Heavy Metal Concentrations in the Vegetables

The concentrations of heavy metals in vegetables from the study area and Chinese maximum allowable concentration (MAC) of each heavy metal in the vegetables (GB 2762-2017) are presented in Table 2. The As, Cd, Cu, Pb, and Zn concentrations [range (mean)] in the vegetables were 0.02–1.33 (0.45), 0.01–1.34 (0.33), 0.12–3.01 (1.12), 0.10–1.47 (0.61), and 1.11–9.36 (4.27) mg/kg, respectively. The concentrations of heavy metals in vegetables were much lower than the results found by Li et al. [31], who reported that the As, Pb, Cd, Cu, and Zn concentrations in the edible parts of vegetables collected around Jijie Smelter in Gejiu, China, were 2.47, 3.69, 0.46, 1.65, and 7.88 mg/kg, respectively. This difference between our results and those of Li et al. [31] could be attributed to high concentrations of heavy metals in soils in the previous study. In this earlier study, the concentrations of As, Cd, Cu, Pb, and Zn in soils around Jijie smelters were 390.54, 9.10, 310.50, 893.36, and 611.89 mg/kg, respectively. In addition, the concentrations of Pb and Zn in vegetables in this study were obviously lower than those reported around a Pb/Zn smelter in Hunan Province, China, except for Cd [24]. However, the concentrations of As, Cd, Cu, and Pb in vegetables were significantly higher than those around a Zn smelter in Huluodao, China [11]. Compared with maximum allowable concentrations (MACs) (GB2762-2017), 38.14%, 57.73%, and 52.58% of the vegetable samples exceed their corresponding MACs for As, Cd and Pb, respectively.

The concentrations of As, Cd, Cu, and Zn decreased in the order of leafy, rootstalk, legume, and fruit vegetables (Table 2), which was consistent with results from Zhou et al. [23], Obiora et al. [43], and Qureshi et al. [44]. There were no significant differences between the leafy and rootstalk vegetables for As and Cd in the present study (*p* > 0.00). However, Chen et al. [45] reported that the concentration of Cd in leafy vegetables was nearly three times that in rootstalk vegetables. Vegetables could readily absorb Cd from soils through their roots because of the high bioavailability of Cd [46]. The Pb concentrations in leafy vegetables were 5.16, 2.64, and 4.45 times those in legume, rootstalk, and fruit vegetables in this study. Similarly, Li et al. [24] reported that the Pb concentration in leafy vegetables was about twice that in non-leafy vegetables. Because of their large surface areas, leafy vegetables can readily assimilate atmospheric particles containing heavy metals through their stomatal pores and the cuticle [9]. Moreover, transpiration plays an important role in the accumulation of heavy metals in plants. Gao et al. [47] reported that heavy metals in soils could be readily absorbed by the roots of leafy vegetables because of high transpiration and translocation rates. Moreover, we will study the heavy metals in the soils and foodstuff at the distance from the smelters, and the regression curve will be analyzed to study the influence of the smelter activities. Therefore, vegetables, particularly leafy vegetables, around the Pb/Zn smelter are seriously contaminated with As, Cd, and Pb, and more attention should be given to the potential health risks for local residents via vegetable consumption.

### 3.3. BCFs of Heavy Metals in Vegetables

**Figure 2:**
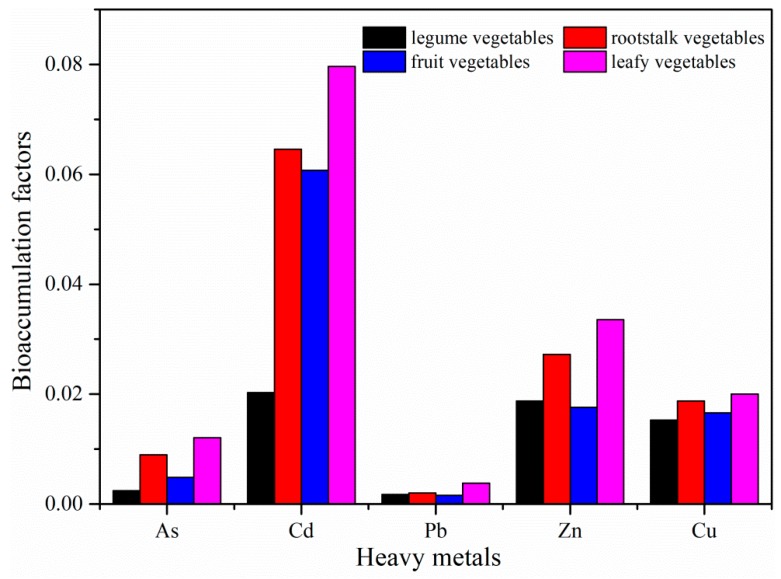
Bioaccumulation factors of heavy metals in different vegetables.

The BCFs were used to evaluate the abilities of the vegetables to accumulate heavy metals. The BCFs of heavy metals in different vegetables are shown in Figure 2. The BCFs of As and Cd were in the order of leafy > rootstalk > fruit > legume vegetables, and the BCFs of Cu, Pb, and Zn were in the order of leafy > rootstalk > legume > fruit vegetables. The average BCFs of heavy metals in the vegetables decreased in the order of Cd (0.055), Zn (0.024), Cu (0.018), As (0.007), and Pb (0.002). These results show that Cd is easily transferred from soil to the edible parts of vegetables, which agrees with the results from other studies [11,31]. The high accumulation ability of Cd may be ascribed to its bioavailability in soils and efficient transport rate in vegetables. Cd can enter plant root cells by occupying Zn^2+^ or Ca^2+^ channels and then combining with transport proteins [48]. By contrast, Pb in the soils produces an insoluble chelate with soil organic matter, and cannot be readily absorbed by plants. Wang et al. [49] reported that Pb was poorly translocated from soil to vegetables. Cu and Zn will be easily absorbed by vegetables because of their functions as plant micronutrients, resulting in high BCFs for these metals in vegetables. Therefore, the accumulation ability of heavy metals varies among the vegetables, which may be affected by soil physicochemical properties and the different morphologies/physiologies of the vegetables.

In addition, high BCFs of Cd were found in pakchoi, cauliflower, carrot, and white radish. These are typical cruciferous vegetables, and have a strong ability to accumulate Cd from soils. However, low concentrations of Cd were found in cabbage, which is also a cruciferous vegetable. These results showed that the Cd accumulation ability in cruciferous vegetables can vary, even within the same genus.

### 3.4. The EDIs of Heavy Metals

**Figure 3:**
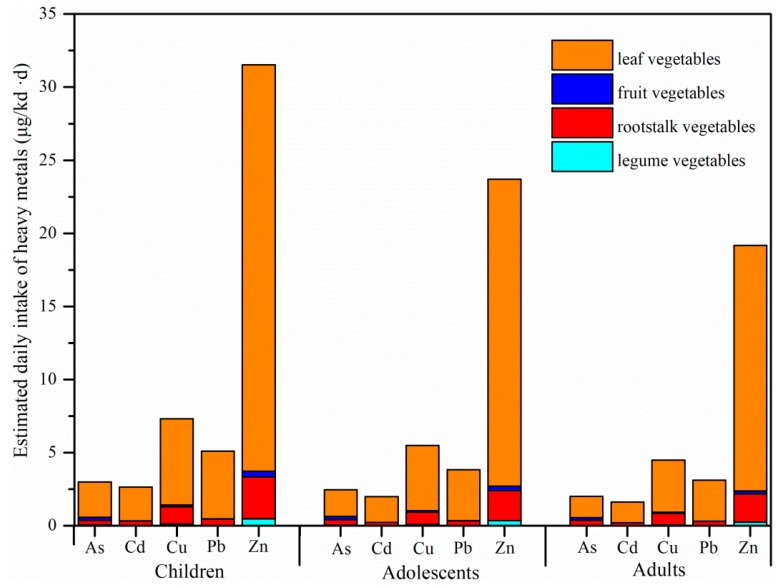
Daily intakes of heavy metals for children, adolescents, and adults.

The EDIs of heavy metals for children, adolescents, and adults are shown in Figure 3. Among the different populations, the highest heavy metal EDIs were found in children, with means of 2.99, 2.64, 7.32, 5.11, and 31.53 μg/(kd·d) for As, Cd, Cu, Pb, and Zn, respectively, followed by adolescents (As: 2.46 μg/(kd·d); Cd: 1.99 μg/(kd·d); Cu: 5.48 μg/(kd·d); Pb: 3.84 μg/(kd·d); Zn: 23.71 μg/(kd·d)) and adults (As: 2.02 μg/(kd·d); Cd: 1.61 μg/(kd·d); Cu: 4.48 μg/(kd·d); Pb: 3.11 μg/(kd·d); Zn: 19.18 μg/(kd·d)). The EDIs of heavy metals via vegetable consumption for the different populations decreased in the order of Zn > Cu > Pb > As > Cd.

The Cd EDIs for different populations via vegetable consumption in this study were lower than those in industrial areas of Huludao [11], Xiangtan County [45], and Daye [10] in China. However, the Cd EDIs were much higher than the results studied by Li et al. [24], who found that the EDIs of Cd via vegetable consumption were 1.46 and 1.25 μg/kd·d for adults and children, respectively, around a Pb/Zn smelter in Zhuzhou, China. The Cu EDIs in the present study were significantly lower than that for local residents around Cu smelters in Guixi [4] and Dexing [50] in China. The Cd EDIs via vegetable consumption in the present study were significantly higher than that in Europe (0.25 μg/kd·d) [51]. For As, the adult EDI was higher than those in France (0.1 μg/kd·d) and Canada (0.29 μg/kd·d), but lower than that in Bangladesh (3.0 μg/kd·d) [52].

Provisional tolerable daily intakes (PTDIs) of heavy metals have been established for food safety. The PTDIs of As, Cd, Cu, and Zn set by the Food and Agriculture Organization of the United Nations and World Health Organization (FAO/WHO) are 3.0, 0.83, 50, and 1000 μg/kd·d, respectively [52,53,54]. The PTDIs of Pb for adults and adolescents are 1.5 and 0.5 μg/kg·d, respectively [55]. The EDIs of As, Cd, and Pb for different populations in the present study were higher than the corresponding PTDIs. This suggests that adverse effects occur for local residents near the Pb/Zn smelters. By contrast, the Cu and Zn EDIs in the present study were lower than the corresponding PTDIs.

The contribution of vegetable consumption to the EDIs of heavy metals in this study was not calculated because of a lack of data for heavy metal concentrations in other foodstuff. However, consumption of staple foods and vegetables are the main sources of heavy metals for local residents in South China. For example, vegetables were the largest contributor to the EDIs of Zn, Pb, and Cu for residents near a Cu smelter in Guixi, China [56]. Similar results were found by Yu et al. [57] who reported that vegetable consumption accounted for 31.3% to 67.8% of the EDIs of Pb in mining and smelting areas in Jiangxi Province, China. Therefore, it is necessary to take effective measures to reduce heavy metal concentrations in vegetables to prevent the exposure of local residents.

### 3.5. Probabilistic Health Risk Assessment

The non-carcinogenic risks from vegetable consumption for children, adolescents, and adults were assessed using the THQs and HIs. Considering the variation of heavy metals in vegetables and attributes of the different populations, the non-carcinogenic risks were simulated using the Monte Carlo method. The 5th, 25th, 50th, 75th, and 95th percentile values of the distributions of the THQs of heavy metals for children, adolescents, and adults are shown in Table 3. The THQs of As for children, adolescents, and adults were higher than one even at the 5th percentile, suggesting that most of the residents were exposed to a significant health risk from vegetable consumption. For Cd, the 50th percentile of THQs slightly exceeded or approached one for children, adolescents, and adults, indicating that about 50% of people experienced non-carcinogenic risks from dietary intake of Cd. For Pb, the 75th percentiles of the THQs were close to one, with THQs of 0.97, 0.89, and 0.77 for children, adolescents and adults, respectively, which suggested that nearly 25% of the people may have health risks from vegetable consumption. The percentile values for the THQs for Cu and Zn via vegetable consumption were all less than one in all the different populations, indicating negligible risks to local residents from these metals.

The non-carcinogenic risks of heavy metal exposure for the different populations were in the order of As > Cd > Pb > Cu > Zn. Although the percentage of vegetable samples contaminated with As was the lowest among the heavy metals, the non-carcinogenic risk of As was higher than that of other heavy metals such as Cd, Pb, Cu, and Zn. Similar results were also found for the THQs of As via vegetable consumption in a previous study [38]. These results indicate that humans are vulnerable to As exposure. The THQs of Cd for children, adolescents, and adults were higher than the results from Chen et al. [45], who reported 90th percentile THQs of Cd of more than one for leafy vegetable consumption in Xiangtan County, China. Similarly, the THQs for As, Cd, Cu, Pb, and Zn were significantly higher than those from abandoned copper mines in Korea [58]. However, the THQs of Pb via vegetable consumption for local residents were significantly lower than those from a large scale study near a Pb/Zn smelter in Zhuzhou, China [24]. Although there was a lack of data for the dose–response relationships of As, Cd, and Pb in the population in the study area, high THQs for As, Cd, and Pb in the present study showed a significant probability of heavy metal-induced disease for local residents. Moreover, the 5th, 25th, 50th, 75th, and 95th percentiles of the THQs of heavy metals for children were higher than those for adolescents and adults, indicating that children are exposed to significant potential risks via vegetable consumption. Overall, As, Cd, and Pb can be regarded as the priority contaminants because of their high non-carcinogenic risks.

**Figure 4:**
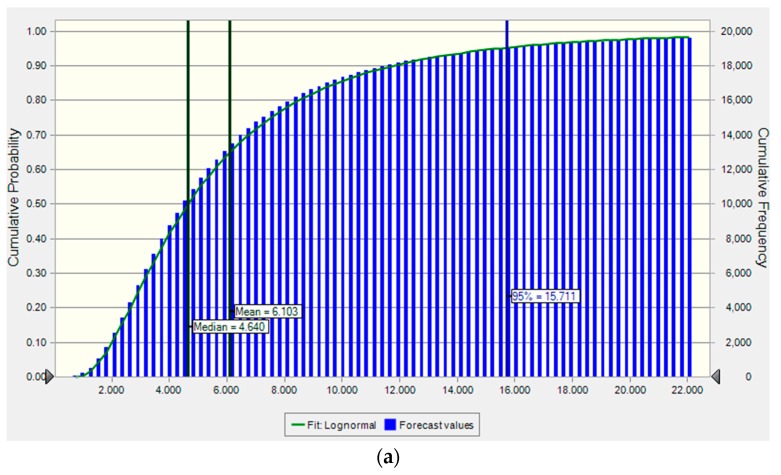

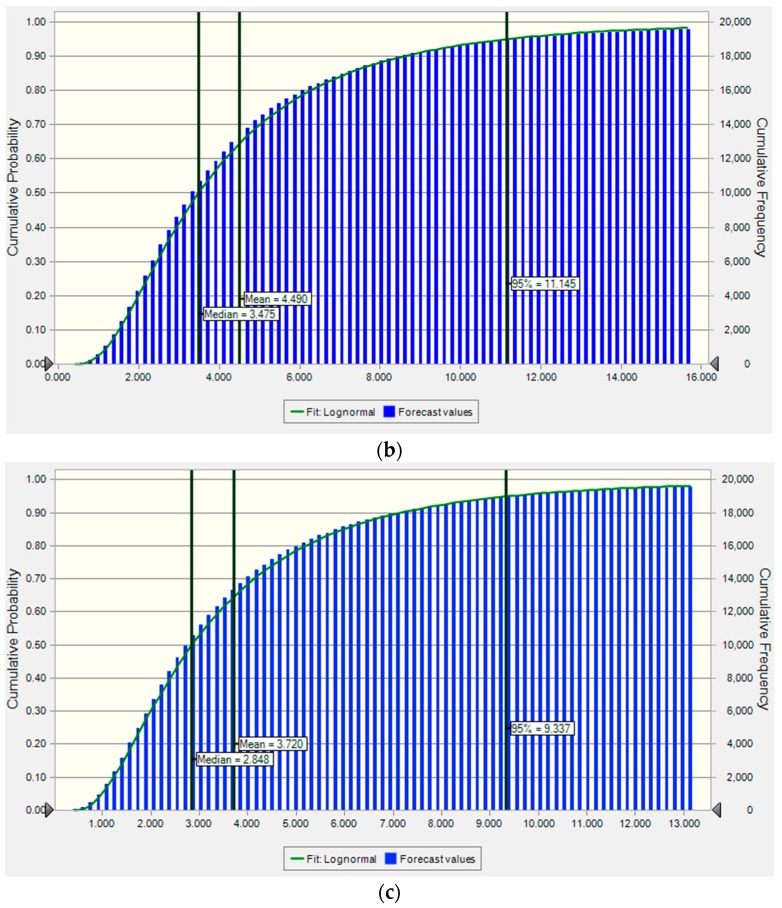
Cumulative distribution of the hazard indexes (HIs) of heavy metals for (**a**) children, (**b**) adolescents, and (**c**) adults.

HIs were calculated using the sum of THQs of individual heavy metals. The cumulative probabilities of the HIs via vegetable consumption for children, adolescents, and adults using a Monte Carlo simulation are shown in Figure 4. The median HIs for children (4.64), adolescents (3.48), and adults (2.85) suggest that there were high potential non-carcinogenic risks to the residents from cumulative exposure. The 95th percentiles of the HIs via vegetable consumption for children, adolescents, and adults were 15.71, 11.15, and 9.34, respectively, indicating potential risks for all age groups. Children were more vulnerable to dietary heavy metal intake, which may be ascribed to their higher metabolisms and absorption capacities compared with adolescents and adults [59].

### 3.6. Sensitivity Analysis

**Figure 5:**
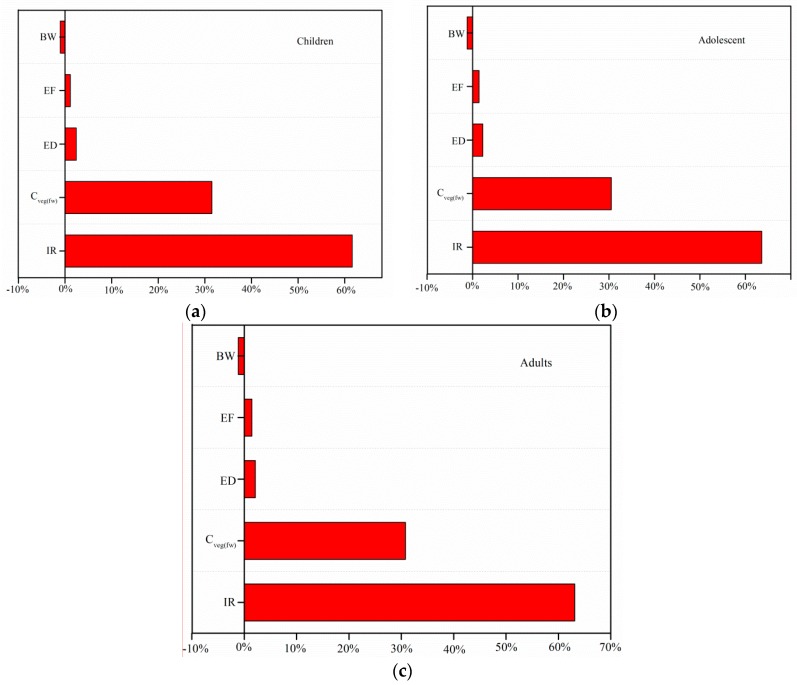
Sensitivity analysis of non-carcinogenic risks for (**a**) children, (**b**) adolescents, and (**c**) adults.

Tornado plots were plotted to show the sensitivity analysis using a percentage scale for the Spearman correlation coefficient of each input variable (Figure 5). The most influential variable for risk assessment was the IR, with contributions of 61.6%, 63.6%, and 63.1% for children, adolescents, and adults, respectively. The concentration of heavy metals in vegetables (*C_veg_*) was the second most influential variable for the risk assessment, with contributions of 31.5%, 30.5%, and 30.8% to the output variable for children, adolescents, and adults, respectively. The ED, EF, and BW for different populations were less influential on the output of the risk assessment. Therefore, the IR and concentration should be investigated to improve the accuracies of the probability distributions of these critical variables in the risk assessment.

### 3.7. Uncertainty Analysis

The reliability of the probabilistic risk assessment depends on an appropriate probability distribution of exposure parameters, such as *C_veg_*, *IR*, *ED*, *EF*, and *BW*, in the risk assessment model. To reduce the risk assessment error, the probability distributions of *C_veg_*, *IR*, *ED*, *EF*, and *BW* were developed using data from the survey in this study. The RfD data was obtained from the USEPA because the limited toxicity data available for humans in China might not reflect the true toxic responses of humans to heavy metals [60]. Moreover, spatial variation of the exposure variables was not considered in the probabilistic risk assessment [29]. Further studies should analyze the probabilistic human risk from a spatial perspective.

An effective risk assessment method considers the bioavailability of heavy metals [61]. The bioavailability ranges of Cd, Cu, Pb, and Zn in vegetables have previously been measured at 21–96%, 20–68%, 30–77%, and 69–94%, respectively [61]. Moreover, the bioavailability concentrations of heavy metals to humans vary among individuals because of differences in ingestion rates and body weight. Therefore, the bioavailability concentrations of heavy metals should be taken as the real exposure dose in the risk assessment or the results will be unreliable. In this study, we assumed 100% bioavailability of the heavy metals in the vegetables, resulting in overestimation of the non-carcinogenic risk to some degree.

Finally, the interaction (antagonistic or synergistic) of multiple heavy metals was ignored in this study. The total non-carcinogenic risks of heavy metals were calculated using the sum of risks from individual heavy metals in this study, which may have underestimated or overestimated the human health risk. Overall, despite the uncertainty and limitations in the probabilistic health risk assessment, the results of this study provide valuable information for the environmental management of the risks to local residents.

## 4. Conclusions

Serious contamination of soils and vegetables with As, Cd, Cu, Pb, and Zn was found around the Pb/Zn smelters. Approximately 51.67%, 100%, 53.87%, 62.5%, and 52.25% of the soil samples had As, Cd, Cu, Pb, and Zn concentrations that exceeded the corresponding screening values, respectively. Leafy vegetables were more seriously contaminated with heavy metals than legume, rootstalk, and fruit vegetables. In vegetables, Cd had the highest BCF, and this was followed by Zn, Cu, As, and Pb. We concluded that the heavy metals As, Cd, and Pb are the primary contributors to non-carcinogenic risks via vegetable consumption. The HI results (median and 95th percentile, respectively) were 4.64 and 15.71 for children, 3.48 and 11.15 for adolescents, and 2.85 and 9.34 for adults, which suggested there were significant health risks to local residents, especially children. Therefore, effective strategies for heavy metal control in surrounding areas should be developed to reduce heavy metal exposure and health risks to local residents.

## Figures and Tables

**Table 1:** Physicochemical characteristic of the soils from the study area (mg/kg).

	pH	SOM	As	Cd	Cu	Pb	Zn
Min	5.56	28.31	17.24	0.38	15.21	23.24	16.21
Max	7.26	70.45	165.42	11.40	349.87	609.49	939.83
Media	6.24	42.65	86.52	2.55	105.17	122.2	158.69
Mean	6.43	45.47	116.76	3.59	158.56	196.96	236.74
SD	0.78	12.32	63.73	4.22	101.67	165.11	188.98
CV(%)	11.95	27.09	54.58	117.50	64.12	83.83	79.83
S-Wp	1.38	0.98	0	0	0	0	0
RSV	-	-	40	0.3	50	90	200

SOM: soil organic matter. Min: minimum. Max: maximum. SD: standard deviation. CV: coefficient of variation. S-Wp: significance level of the Shapiro-Wilk test for normality. SV: risk screening values for heavy metals.

**Table 2:** Concentrations of heavy metals in different vegetables (mg/kg, *fw*).

Samples	Parameter	As	Cd	Cu	Pb	Zn
Leafy vegetables	Min	0.03	0.03	0.12	0.24	3.61
	Max	1.33	1.34	1.89	1.47	9.36
	Media	0.49	0.40	1.42	0.99	5.50
	Mean	0.52a	0.39a	1.26a	0.98a	5.94a
	SD	0.39	0.34	0.52	0.35	1.72
MAC		0.5	0.2	-	0.3	-
Rootstalk vegetables	Min	0.23	0.02	0.41	0.10	1.11
	Max	0.78	0.72	3.02	0.88	8.12
	Media	0.42	0.39	0.92	0.27	4.37
	Mean	0.51a	0.36a	1.03ab	0.37b	4.51b
	SD	0.14	0.21	0.64	0.25	1.24
MAC		0.5	0.1	-	0.1	-
Legumes vegetables	Min	0.06	0.02	0.45	0.11	1.35
	Max	1.09	0.33	1.52	0.31	6.49
	Media	0.26	0.06	0.85	0.16	3.72
	Mean	0.41b	0.13b	0.97b	0.19c	3.75bc
	SD	0.44	0.12	0.44	0.09	1.71
MAC		0.5	0.1	-	0.2	-
Fruit vegetables	Min	0.02	0.01	0.70	0.14	2.19
	Max	0.56	0.12	1.63	0.30	3.98
	Media	0.05	0.04	0.88	0.23	3.17
	Mean	0.14c	0.05c	0.96b	0.22c	3.13c
	SD	0.20	0.04	0.34	0.06	0.68
MAC		0.5	0.05	-	0.1	-
Total vegetables	Min	0.02	0.01	0.12	0.10	1.11
	Max	1.33	1.34	3.01	1.47	9.36
	Media	0.41	0.21	0.99	0.42	4.07
	Mean	0.45	0.33	1.12	0.61	4.27
	SD	0.33	0.31	0.54	0.44	2.15
	Exceeding (%)	38.14	57.73	-	52.58	-

MAC: maximum allowable concentration (GB2762-2017). Different letters (a, b, and c) in the same column indicate a significant difference at *P* < 0.05.

**Table 3:** Target hazard quotients (THQs) of different populations in the study area.

Population		THQ				
	Probability	As	Cd	Cu	Pb	Zn
Children (4–11 years)	5%	1.76	0.28	0.06	0.26	0.03
	25%	3.21	0.58	0.08	0.42	0.04
	50%	5.02	1.20	0.12	0.63	0.05
	75%	7.95	1.73	0.16	0.97	0.08
	95%	12.19	3.71	0.26	1.89	0.13
Adolescent (12–17 years)	5%	1.71	0.16	0.05	0.24	0.03
	25%	3.02	0.53	0.07	0.39	0.04
	50%	4.95	0.98	0.10	0.58	0.06
	75%	7.67	1.68	0.14	0.89	0.09
	95%	11.23	3.10	0.23	1.74	0.12
Adults (>18 years)	5%	1.25	0.24	0.04	0.20	0.02
	25%	2.73	0.51	0.07	0.34	0.03
	50%	4.33	0.88	0.09	0.50	0.04
	75%	6.86	1.51	0.12	0.77	0.06
	95%	10.33	3.28	0.20	1.52	0.11

## Supplementary Materials

The following are available online at https://www.mdpi.com/1660-4601/16/18/3267/s1, Table S1: Distribution of each parameter for different population groups in Monte Carlo simulation. Table S2: Pearson’s correlation of the heavy metals in the soils. Table S3: The results of principal component analysis for heavy metals in the soils.
