# Supplementary material for: Probabilistic Human Health Risk Assessment of Heavy Metal Intake via Vegetable Consumption around Pb/Zn Smelters in Southwest China
Source: Int J Environ Res Public Health. 2019 Sep 5;16(18):3267. doi: 10.3390/ijerph16183267 (PMC6765770; doi:10.3390/ijerph16183267)
Supplement: Supplementary file 1 [file ijerph-16-03267-s001.pdf]

Table S1. Distribution of each parameter for different population groups in Monte Carlo simulation.

**Symbol**	parameter	unit	element	population	PDFs	Distribution
$C_{veg(fw)}$	Concentrations of heavy metal in vegetables	mg/kg (fw)	As		0.30±1.07	Log-normal
			Cd		0.20±1.02	Log-normal
			Cu		0.98±0.56	Log-normal
			Pb		0.45±0.85	Log-normal
			Zn		3.74±0.55	Log-normal
EF	Exposure frequency	day/year			352(300-365)	Triangle
ED	Exposure duration	year		children	0-6	Uniform
				young	6-18	Uniform
				adults	18-60	Uniform
AT	Average time	d			ED×365	Point
IR	Ingestion rate	g/d (fw)		children	90.59±1.08	Log-normal
				young	169.42±1.07	Log-normal
				adults	203.34±1.18	Log-normal
BW	Body weight			children	21.98±1.19	Log-normal
				young	40.12±1.20	Log-normal
				adults	56.84±1.09	Log-normal
RfD	Reference dose	μg/(kg BW·d)	As		0.3	Point
			Cd		1	Point
			Cu		40	Point
			Pb		3.57	Point
			Zn		300	Point

Geometric mean ± geometric standard deviation for Log-normal distributions; Minimum-maximum for the uniform distributions. Mean(Minimum-maximum) for triangle distributions. fw：fresh weight.

Table S2. Pearson's correlation of the heavy metals in the soils.

	**As**	**Cd**	**Cu**	**Pb**	**Zn**
As	1	0.381	0.577*	0.413*	0.480*
Cd		1	0.425*	0.584*	0.477*
Cu			1	0.296	0.229
Pb				1	0.929*
Zn					1

Table S3. The results of principal component analysis for heavy metals in the soils.

**Component**	Initial eigenvalues			Extraction sums of squared loadings			Rotation sums of squared loadings		
	Total	% of variance	Cumulative (%)	Total	% of variance	Cumulative (%)	Total	% of variance	Cumulative (%)
**Total variance explained**									
**1**	2.573	51.458	51.459	2.573	51.458	51.458	2.273	45.454	45.454
2	1.720	34.410	85.868	1.720	34.410	85.868	2.021	40.414	85.868
3	0.684	13.679	99.546						
4	0.018	0.369	99.916						
5	0.004	0.084	100.00						

Metal	Component matrix		Rotated component matrix	
	PC1	PC2	PC1	PC2
Pb	0.778	-0.577	0.969	-0.002
Zn	0.809	-0.535	0.968	0.050
Cd	0.640	-0.163	0.612	0.248
Cu	0.680	0.724	0.118	0.986
As	0.664	0.743	0.093	0.992
